# Lectures on Aural Catarrh; or the Commonest Forms of Deafness and Their Cure

**Published:** 1872-06

**Authors:** 


### Books Reviewed.

*Lectures on Aural Catarrh; or the commonest forms of Deafness and their cure.* By Peter Allen, M. D., etc. New York: Wm. Wood & Co. 1872.

The author has presented us in this little work twelve lectures on the subject of Aural Catarrh, which we have no doubt will find a welcome reception by every physician, into whose hands it may chance to fall. The subject of Aural Catarrh is one that is but briefly spoken of in most of the text books to be found in the libraries of the general practitioner, and the monograph of Dr. Allen will supply a deficiency long felt by physicians, who have not had access to the more extended treatises on aural difficulties, and yet who are often called upon to treat this class of diseases.

The hope of the author that it may be of help in telling the physician "how to examine, what to look for, and where to find the disease, in a case of aural catarrh," is we think fully realised, and we look upon his work as a valuable guide to the general practitioner.

